# Editorial: Brain metastasis and systemic target therapy: implications for neurosurgeons

**DOI:** 10.3389/fsurg.2026.1761513

**Published:** 2026-01-19

**Authors:** Cleiton Formentin, Maira Cristina Velho, Erion Junior de Andrade, Guilherme Finger

**Affiliations:** 1 Department of Neurology, State University of Campinas, Campinas, Brazil; 2 Hospital Sirio-Libanes, São Paulo, Brazil; 3 Department of Neurology, Universidade Federal do Rio Grande do Sul, Porto Alegre, Brazil; 4 Department of Neurosurgery, Universidade Federal de Ciencias da Saude de Porto Alegre, Porto Alegre, Brazil; 5 Department of Neurological Surgery, Indiana University Indianapolis, Indianapolis, IN, United States

**Keywords:** brain metastases, neuro oncology, neurosurgeon, precision oncology, radio-oncology, systemic therapies

Brain metastases continue to represent one of the most complex and demanding challenges for neurosurgeons and neuro-oncologists. Advances in systemic therapies have significantly prolonged survival across multiple cancer types, but this improved longevity has reshaped the neurosurgical landscape: more patients now live long enough to develop intracranial disease that requires tailored surgical, radiosurgical, or multimodal intervention. The Research Topic “*Brain Metastasis and Systemic Target Therapy: Implications for Neurosurgeons*” was conceived to highlight precisely this interface—how evolving systemic treatments (including targeted therapies, immune checkpoint inhibitors, and molecularly driven strategies) are influencing neurosurgical decision-making, timing, complications, and long-term outcomes.

The objective of this collection is both timely and multifaceted. It aims to explore how systemic therapies affect surgical candidacy and outcomes; to address how neurosurgeons should incorporate the molecular diagnosis of intracranial disease—particularly the documented divergence between primary tumors and their brain metastases—into clinical reasoning; to evaluate how surgical or radiosurgical timing should be coordinated with systemic regimens; and to examine therapy-related complications or neurotoxicity with direct implications for operative planning. Ultimately, this collection strives to move beyond the traditional divide between “neurosurgery for brain metastases” and “systemic oncology for metastases”, toward a collaborative, fully integrated paradigm. The four original contributions in this Topic approach this paradigm shift from distinct yet complementary angles.

The first article examines molecular divergence between primary tumors and metastatic brain lesions, demonstrating a high frequency of discordance and underscoring its implications for neurosurgical sampling and planning (Sun et al.). In the precision-oncology era, the neurosurgeon is no longer merely a remover of mass lesions but a strategic partner in molecular pathway interrogation. This study reinforces the dual purpose of neurosurgical intervention: cytoreduction and procurement of high-quality tissue for next-generation sequencing, which can meaningfully alter systemic management.

A second contribution employs large-scale population-based data to develop a prognostic nomogram for patients with brain metastases from renal cell carcinoma (Wang et al.). By integrating demographic and clinical parameters, this work illustrates how systemic disease context and neurosurgical decisions must be evaluated through individualized risk assessment—an approach increasingly central to oncology.

The remaining two contributions are case reports that reflect the expanding integration between systemic therapy and surgical management in thoracic oncology. One details a patient with lung adenocarcinoma achieving pathological complete response following conversion therapy before resection (Ran et al.). The other highlights long-term survival in a patient with lung squamous cell carcinoma with high PD-L1 expression and elevated tumor mutational burden, managed through immune-chemotherapy followed by radical surgery (Xu and Ma). Both cases demonstrate how effective systemic control can generate new surgical opportunities, emphasizing the necessity of close coordination among oncologists, neurosurgeons, and radiation specialists.

Collectively, these studies point toward a central conclusion: the neurosurgical management of brain metastases can no longer be viewed as a linear sequence—“remove lesion, relieve mass effect, refer for radiotherapy”. Instead, neurosurgeons must integrate into their decision-trees:

systemic disease status and projected trajectory;
molecular and immunologic profiles of primary and metastatic lesions;
optimal timing of surgery or radiosurgery relative to systemic therapy;
unique risk considerations associated with immunotherapy and targeted agents; and
strategies for sustained intracranial control within the context of prolonged survival.

More broadly, this shift echoes trends across modern oncology, where metastatic disease increasingly resembles a chronic, manageable condition. The brain is no longer merely a palliative site, but a domain requiring sophisticated, multidisciplinary intervention. For neurosurgeons, this evolution demands increasing fluency in systemic therapies, molecular diagnostics, and multimodal treatment timing. The neurosurgeon becomes a central strategic agent in a therapeutic ecosystem in which intracranial disease cannot be dissociated from systemic cancer biology.

Yet significant gaps persist. Prospective studies incorporating both neurosurgical and systemic oncologic endpoints are urgently needed, as are standardized algorithms for surgical timing and evidence-based frameworks for integrating molecular findings into operative planning. Furthermore, the issue of molecular discordance between primary tumors and metastatic sites requires sustained investigative and clinical attention, given its profound implications for personalized therapy.

**Figure 1:**
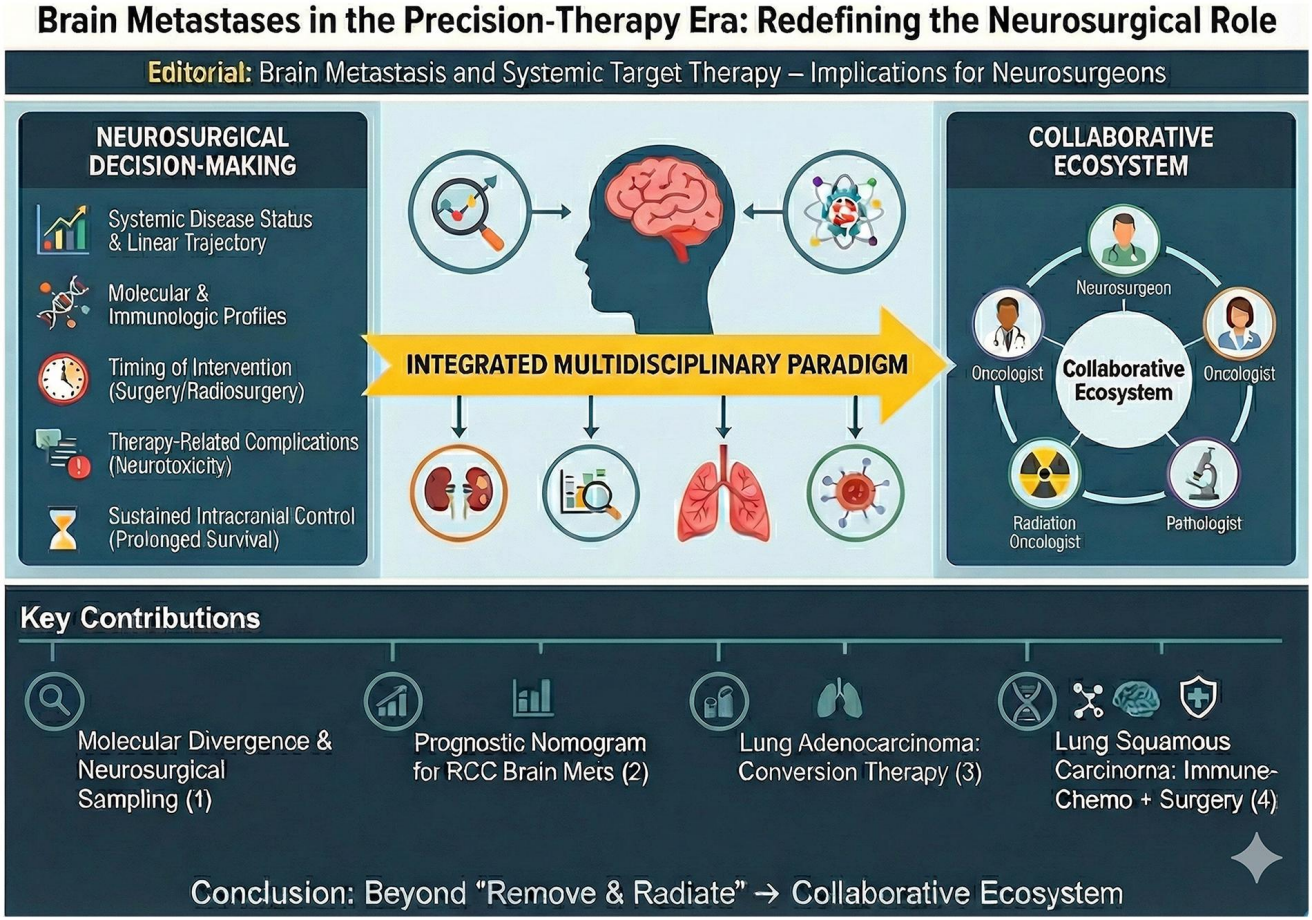
Graphical abstract: a flat-vector infographic summarizing how advances in systemic targeted therapies reshape the neurosurgical management of brain metastases.

In conclusion, this Research Topic represents an important step toward deeper integration of systemic therapy and neurosurgical practice for the management of brain metastases (Figure 1). The contributions collectively reinforce that neurosurgery must adapt to the precision-oncology era—defined by genomic profiling, immunomodulation, and multidisciplinary collaboration. Neurosurgeons are now indispensable navigators in a rapidly evolving therapeutic landscape where intracranial disease is inseparable from systemic cancer progression and biology.

